# Differentiating between hospitals according to the “maturity” of quality improvement systems: a new classification scheme in a sample of European hospitals

**DOI:** 10.1136/qshc.2008.029389

**Published:** 2009-01-26

**Authors:** M J M H Lombarts, I Rupp, P Vallejo, N S Klazinga, R Suñol

**Affiliations:** 1 Academic Medical Center, Department of Social Medicine, University of Amsterdam, Amsterdam, the Netherlands; 2 Avedis Donabedian Institute, Autonomous University of Barcelona, Barcelona, Spain

## Abstract

**Aim:**

This study, part of the Methods of Assessing Response to Quality Improvement Strategies (MARQuIS) research project focusing on cross-border patients in Europe, investigated quality policies and improvement in healthcare systems across the European Union (EU). The aim was to develop a classification scheme for the level of quality improvement (maturity) in EU hospitals, in order to evaluate hospitals according to the maturity of their quality improvement activities.

**Methods:**

A web-based questionnaire survey designed to measure quality improvement in EU hospitals was used as the basis for the classification scheme. Items included for the development of an evaluation tool—the maturity index—were considered important contributors to quality improvement. The four-stage quality cycle (plan, do, check and act) was used to determine the level of maturity of the various items. Psychometric properties of the classification scheme were assessed, and validation analyses were performed.

**Results:**

A total of 389 hospitals participated in a questionnaire survey; response rates varied by country. For a final sample of 349 hospitals, it was possible to construct a quality improvement maturity index which consisted of seven domains and 113 items. The results of independent analyses sustained the validity of the index, which was useful in differentiating between hospitals in the research sample according to the maturity of their quality improvement system (defined as the total of all quality improvement activities).

**Discussion:**

Further research is recommended to develop an instrument which for use in the future as a practical tool to evaluate the maturity of hospital quality improvement systems.

The acid test for any quality improvement (QI) system is its impact on the quality of patient care. Measuring such impact reliably, however, is difficult, and studies presenting significant measurable gains that can be attributed to hospital-wide QI systems are rare. Most studies have reported what hospitals actually do to assure and improve quality. Over the past two decades, several projects at the European level have set out to characterise quality assurance and QI activities in hospitals. The Concerted Action Programme on Quality Assurance in Hospitals (COMAC/HSR/QA), carried out in 1990–3 in 15 European countries, was one of the first to report on the application of quality assurance methods in European hospitals.^1^ As a follow-up, the European Union (EU) funded the 4-year ExPeRT project beginning in 1996, which shifted the focus to mechanisms of external assessment, catalogued the range of programmes offering external evaluation, and described their use in assessing and implementing QI systems.^2^ Because no concise, comprehensive measures are available as a gold standard for QI implementation, more recent studies have tried to define developmental stages of QI systems, for either external comparative reasons or for hospital self-assessment.^3^^–^5 In the Methods of Assessing Response to Quality Improvement Strategies (MARQuIS) study we aimed at moving to the next level of analysis, and tried to evaluate the impact of hospital-wide QI strategies on quality activities and outputs, since this has not been done in previous research at the European level. To facilitate our analysis we developed a classification model based on the maturity of hospital QI systems, defined as the total set of QI activities performed. This classification model was named the quality improvement maturity index. Its design and application to the classification hospitals in our research sample are the focus of this article.

This study was conducted as part of the MARQuIS project, funded as part of the Scientific Support to Policies component of the EU 6th Framework Research Programme. The MARQuIS project aims to investigate and compare different QI policies and strategies in healthcare systems across the 25 member states of the EU, and to consider their potential use when patients cross borders to receive healthcare.

## METHODS

### Design of the questionnaire on quality improvement strategies

We conducted a web-based questionnaire survey among acute care hospitals in eight EU member states.^6^ The questionnaire measured QI, defined as the application of quality policies and procedures, quality governance structures, and quality activities to close the gap between current and expected levels of quality. Items for inclusion were selected on the basis of internationally accepted evaluations as contributors to QI. Several sources were consulted, such as existing QI questionnaires,^3^ ^7^^–^11 a review of the quality literature,^11^^–^13 an analysis of accreditation manuals,^14^ ^15^ and the results of previous MARQuIS studies, including a literature review covering QI strategies in EU member states.^16^^–^18 The questionnaire consisted of four sections: one focused on QI at the hospital-wide level, and the other three dealt with specific medical conditions. These conditions were selected based on two criteria: the condition had to represent a significant volume of cross-border patient care,^16^ and the combination of conditions was intended to cover the most relevant services offered by hospitals—that is, emergency surgical and medical services, and maternal and neonatal services. The limitation to three conditions (acute myocardial infarctions (AMI), acute appendicitis and deliveries) was to allow for more specific and more detailed data collection. In all, 199 items were included in the questionnaire; all but one were closed questions. Answer categories varied from a two-point to a five-point scale, depending on the type of question. The development of the questionnaire is described in detail elsewhere.^6^

### Participation

The countries participating in this study were Spain, France, Poland, Czech Republic, the UK, Ireland, Belgium, and the Netherlands. In all, 483 acute care hospitals located in these eight EU member states visited the online questionnaire, and ultimately 389 returned a completed questionnaire. The resulting study population consisted of public (80%) and private (20%) hospitals, and included university (23.5%), teaching (48.9%), and non-teaching settings (276%). More detailed information on sampling, recruitment and participation is available elsewhere in this supplement.^6^

### Design of the quality improvement maturity index

As described briefly above, our web-based questionnaire elaborated on previous research^3^^–^11 in the field of QI and management. To define the maturity of a hospital QI system (ie, the total of all QI activities performed), we developed a classification model named the quality improvement maturity index based on a selection of items listed in section 1 of the questionnaire, which dealt with hospital-wide policies, procedures, and activities. The QI maturity index consisted of seven domains, totalling 113 items from section 1 of the questionnaire:

1. policy, planning, documents (20 items);
2. leadership (36 items);
3. structure (19 items);
4. general QI activities (8 items);
5. specific QI activities (20 items);
6. patient involvement (6 items);
7. accountability (4 items).

These domains were constructed based on conceptual assumptions, including information from the general part of the questionnaire, and elaborating in part upon previous work by others.^3^ ^7^ Individual items were coded on a four-point scale ranging from 1 (most mature) to 4 (least mature). For items in the questionnaire that used a four-point answer scale (for example: 1 = yes always, 2 =  most of the time, 3 =  sometimes, 4 =  no) the answers could be easily transposed in terms of maturity level, since a lower score already indicated a more favourable answer. However, items using a two-point scale (yes/no) needed to be recoded to fit the four-level maturity index. The recoding procedure for these items was based on the four stages of the quality cycle: plan, do, check and act. All negative answers (ie, answer  = no) were recoded as 4. A positive answer (yes) was assigned a weight based on the principles of the plan-do-check-act cycle, and accordingly re-coded as QI maturity level 1–3 (see table 1).

**Table 1:** Principles for coding and recoding items in the quality improvement (QI) maturity index

Stage of quality improvement maturity level			
1 (Most mature)	2	3	4 (Least mature)
External assessment	Written reports of activities	Policies	No
Internal audits	Implementation of quality and safety activities, ie:	QI plans	
Professionals formally approve policies	Periodic and systematic evaluation of data collected in laboratories	Strategies	
Accountability	Regular calibration or maintenance equipment	Staff responsibility for QI activities	
Feedback to staff and board	Internal budget for QI	Quality or safety teams or committees	
Review and use of indicator data			

To illustrate the nature of the items included, box 1 describes in more detail one of the domains: QI activities. Previous research by Wagner and colleagues yielded a similar domain,^3^ ^7^ since their work was used to design our web-based questionnaire and to formulate the maturity index domains.

**Box 1: Illustration of the construction of one of the seven maturity index domains**

**Questions included in the quality improvement activities domain**

Which of the following internal quality improvement activities take place in your hospital?

- Quality improvement teams or circles
- Internal audit
- Adverse event reporting and analysis
- Risk management and patient safety
- Patient surveys
- Analysis of patient complaints
- Monitoring the views of referring professionals
- Regular staff performance reviews

Answers are scored on a four-point scale:

1 = Yes, this activity takes place systematically in most departments (>50%).

2 = Yes, this activity takes place in most departments (>50%), but not systematically.

3 = Yes, this activity takes place in some departments (<50%).

4 = No, this activity does not take place

### Statistics

The conceptual assumptions underlying the seven domains were globally assessed for each domain separately by factor analysis (principal component analysis, oblimin procedure, forced one-factor solution to determine whether factor loadings globally confirmed the assignment of items to the previously formulated domains), and based on internal consistency reliability (Cronbach α). However, since in this stage of the analysis our main aim was to classify hospitals based on the items selected previously, these results were not used to adjust the domains by excluding items with low factor loadings or items resulting in a higher Cronbach α.

Mean summary scores per domain were computed when the data for at least half of the items were available, or half plus one in the case of uneven numbers. These seven domain mean scores were combined in a mean overall score per hospital, which expressed the institution’s QI maturity level on a scale from 1 (most mature QI system) to 4 (least mature QI system). Again, a requirement for this computation was that data for at least half of the domains had to be available. To further explore the robustness of the QI maturity index, we calculated correlations between the domains (Spearman ρ) as well as the correlation of each domain with the overall QI maturity classification. To validate the QI maturity index, three independent analyses were performed: hypothesis testing; on-site hospital visits; and expert assessment of the maturity of the QI system based on written information.

#### Hypothesis testing

In order to check the validity of our classification scheme, hypothesis testing was done with the self-reported data set to analyse the extremes of the maturity classification—that is, hospitals with the most mature versus least mature QI systems. We tested the relation between QI maturity and selected outputs such as external pressure, compliance with safety requirements and compliance with support of cross-border patients. These outputs were defined by combining items from section 1 of the questionnaire^6^ into a summary score. With the exception of one item, these items were scored on a two-point scale as yes or no,^6^ with lower scores reflecting better outputs. External pressure consisted of nine items:

- use of International Organization for Standardization (ISO) standards;
- European Foundation for Quality Management (EFQM) model for implementing QI strategies;
- external assessment in the preceding 3 years;
- use of peer review (ie, site visits);
- external audit of five laboratories:
-
  - clinical chemistry;
  - pathology;
  - microbiology;
  - pharmacy;
  - diagnostic radiology.

The compliance with safety requirements output consisted of four items:

- standardised, limited number of drugs or a formulary for medications;
- electronic drug prescription system;
- a system for patient identification with bracelets in the emergency department;
- system for patient identification with bracelets for admitted patients.

Lastly, compliance with the support for cross-border patients output included six items:

- formalised arrangement with a translation service for EU patients;
- information leaflets in the other EU languages;
- provision of a case manager for non-native speaking patients;
- one or more designated persons to support foreign EU patients in administrative procedures such as payment or transportation;
- procedure defining how the hospital offers assistance to foreign EU patients in seeking contact with family or friends;
- procedure defining how the hospital offers assistance to foreign EU patients in seeking contact with their family doctor or general practitioner.

Further, to check the association between QI maturity and the service-specific sections of the questionnaire, we tested the use of two performance indicators for the management of AMI patients (section 2 of the questionnaire)—for example, availability in the hospital of the door-to-needle time indicator, and availability of the “use of aspirin within 24 h of AMI diagnosis” indicator.^6^

#### Hospital on-site assessment

Experienced, trained independent external surveyors conducted visits to a selected sample of 89 hospitals that were classified on the basis of their completed questionnaire as having most mature or least mature QI systems. External surveyors were blinded to both the classification status of the hospitals and their questionnaire results. After on-site assessment, the surveyors graded the hospitals from 1 (least developed QI system) to 5 (most developed QI system) based on their own knowledge of hospital QI systems. These grades were compared with the QI maturity index classification using cumulative logit random models. The surveyors also drafted a report for each hospital that included a descriptive summary of the most relevant information, as well as the hospital’s main strengths and weaknesses. The hospital on-site assessment is described in detail elsewhere.^19^

#### Expert assessment of quality improvement system maturity

The reports drafted by the on-site surveyors were analysed by an internationally recognised expert in external quality assessment who classified the hospitals as most mature and least mature based on that information. This expert was blinded to the results of the maturity index classification. The level of agreement between this classification and the maturity index classification was calculated with κ statistics.

## RESULTS

### Statistics

Of the 389 hospitals who responded to the questionnaire, 349 provided enough data to calculate the QI maturity classification. The results of forced one-factor analysis (not shown) showed low (<0.30) to relatively high (0.82) factor loadings, indicating that the domains will require further adjustment and refinement in order to develop our QI maturity classification into a robust instrument.

Internal consistency of the domains was reasonable (Cronbach α  = 0.69) to good (Cronbach α  = 0.89), except for the accountability domain (table 2). For conceptual reasons, however, it was decided to maintain the latter domain in the maturity index. For the combined QI maturity index the Cronbach α was 0.72 (table 2).

**Table 2:** Construction of the quality improvement (QI) maturity index in seven domains and 113 items

Domain	Number of items included	Cronbach α
Policy, planning, documents	20	0.86
Leadership	36	0.89
Structure	19	0.69
QI activities	8	0.75
QI activities (laboratories)	20	0.85
Patient involvement	6	0.82
Accountability	4	0.54
Total	113	0.72

Significant correlations between the different domains was found. Most correlations were statistically significant at the p = 0.01 level (table 3). All correlations were <0.70, indicating that independent aspects were captured by the different domains, except for the correlation between the structure and policy domains, with a correlation of 0.730. Table 3 also shows that all seven domains had notable correlations with the overall QI maturity classification. This means that all domains contributed to the maturity classification; however, the strength of the relationships varied from 0.402 (patient involvement) to 0.766 (QI activities), indicating that further research and development to refine the weightings of the domains might be appropriate.

**Table 3:** Correlations (Spearman ρ) between the domains of the quality improvement (QI) maturity index, and between other domains and the index

	Policy	Leadership	Structure	QI activities	QI activities (laboratories)	Patient involvement	Accountability	QI maturity index
Policy	1.0							0.726*
Leadership	0.469*	1.0						0.686*
Structure	0.730*	0.498*	1.0					0.664*
QI activities	0.564*	0.489*	0.515*	1.0				0.766*
QI activities (laboratories)	0.516*	0.304*	0.499*	0.478*	1.0			0.615*
Patient involvement	0.20*	0.108	0.215*	0.242*	−*0.025*	1.0		0.402*
Accountability	0.238*	0.275*	0.185*	0.257*	0.217*	0.090	1.0	0.553*

*p = 0.01.

### Classification of hospitals

Table 4 summarises the variance in the mean overall QI maturity index score for participating countries. The UK and the Netherlands had lower maximum scores (meaning more mature QI systems) than other countries, but this may have been due to the lower numbers of hospitals from these countries that participated in this study.

**Table 4:** Variance in overall quality improvement maturity classification scores, and distribution of scores

Country	Variance in maturity classification score*		Maturity classification scores (based on quartiles)		
	Min score	Max score	Most mature (⩽25%)	Intermediate maturity (>25%–75%)	Least mature(>75%)
Belgium (n = 24)	2.10	3.10	3	16	5
Czech Republic (n = 38)	1.50	3.00	10	22	8
France (n = 65)	1.87	3.25	11	32	22
Ireland (n = 23)	1.90	3.08	8	12	3
The Netherlands (n = 8)	2.09	2.52	4	4	–
Poland (n = 76)	1.86	3.45	22	33	21
Spain (n = 105)	1.70	3.26	20	55	30
UK (n = 10)	1.77	2.37	9	1	–
Total (n = 349)	1.50	3.45	87	175	87

*Low scores reflect higher maturity.

For the entire sample we categorised hospitals according to their QI maturity level as most mature (⩽25th percentile; n = 87), intermediate (>25th percentile to 75th percentile; n = 175), or least mature (>75th percentile; n = 87). With the exception of the UK and the Netherlands, least mature hospitals were found in all countries, and all countries also had most mature hospitals (table 4).

### Validation

#### Hypothesis testing

The validity of the maturity classification was further explored by analysing the two extreme groups through selected hypothesis testing. Table 5 shows that hospitals with more mature QI systems performed better in all but one of the hypotheses (AMI indicator, aspirin use started within 24 h after AMI).

**Table 5:** Validation of the quality improvement maturity index through hypotheses testing

Hypotheses tested: output was related with maturity of the hospital’s QI system	Operationalisation of values tested		Distribution of QI maturity classification (mean scores)		
	Number of items included(Cronbach α; n)	Significant at p level	Most mature(⩽25%)	Least mature(>75%)	Total
External pressure	9 (0.68; n = 126)	<0.001	1.71 (n = 78)	2.75 (n = 76)	2.25 (n = 310)
Safety*	4 (0.24; n = 313)	<0.001	1.38 (n = 86)	1.53 (n = 85)	1.44 (n = 349)
Support for cross-border care patients	6 (0.61; n = 298)	<0.001	1.60 (n = 86)	1.76 (n = 84)	1.96 (n = 346)
			Distribution of quality improvement maturity classification (%)		
Availability of AMI indicator: door-to-needle time: % yes	1	0.10	65.2	47.3	57.5
Availability of AMI indicator: aspirin <24 h after AMI: % yes	1	0.03	82.4	64.0	71.4

*To avoid overlap contamination in the measures and output, a modified QI maturity index was computed excluding all items related to safety.

#### Hospital site visits and assessment

Table 6 shows the hospital ratings provided by external surveyors. The cumulative logit random effects model showed that hospitals classified as most mature according to the QI maturity index received higher grades, whereas hospitals classified as least according to the QI maturity classification were given worse grades. The odds that the grade would fall below any given category for least mature hospitals were 30.77-fold (95% CI 6.03 to 160.32), the estimated odds for most mature hospitals.

**Table 6:** Agreement between quality improvement (QI) maturity index classification and assessments of independent surveyors*

Evaluator scale	Least mature n (%)	Most mature n (%)
Least developed QI	11 (84.6)	2 (15.4)
	18 (85.7)	3 (14.3)
Intermediate	13 (46.4)	15 (53.6)
	10 (25.0)	30 (75.0)
Most developed QI	0	6 (100)
Total	52 (48.1)	56 (51.9)

*In general, two surveyors audited each hospital. Their responses were included independently in the analysis (either both gave the same response, or each gave a different one). Some hospitals were audited by only one surveyor, and not all of them provided a maturity rating.

#### Expert assessment of quality improvement system maturity

Complete hospital reports were written for 38 of the 89 hospitals that were externally assessed by independent surveyors. The other 51 reports did not include (n = 27), or only partially included (n = 24) the requested summary of main findings, and thus could not be used by the external expert to classify the maturity of the hospitals’ QI systems. Classification by the external expert of the 38 hospitals that were included in the analysis resulted in 76.32% agreement between the expert’s hospital classification and the classification based on the maturity index. The κ value was 0.526 (standard error = 0.128); in cases in which the two evaluations disagreed, the direction of disagreement was positive in 89%.

## DISCUSSION

We constructed a QI maturity index for a sample of European hospitals. The maturity index was found to be useful to differentiate between hospitals according to the maturity of their QI system, defined as the total of QI activities performed. The results of independent analyses sustain the validity of the QI maturity index in our research sample.

Clearly, hospitals with the most mature QI systems were identified in all participating countries. This is in line with a previous MARQuIS study report in which we found that QI strategies were widely applied in all participating European countries.^17^ Considerable variation in the maturity of hospital QI systems was identified both within and between countries, and it is interesting to note that variation within countries seemed to be as high as variation between countries. In future research multilevel analyses may be indicated to unravel the underlying causes of variability within and between countries. It should be stressed that hospitals classified as most mature do not necessarily deliver the best quality care to their patients. However, the hypotheses that we tested to validate the maturity index indicated that maturity of a hospital’s QI system may be positively associated with better outputs.

This study has its limitations. Although data from the questionnaire were self-reported, it has been shown through on-site visits that they seemed to be fairly reliable. Furthermore, selection bias among participating hospitals cannot be ruled out. Although hospitals were sampled randomly, the results need to be interpreted with some caution in terms of generalisability, given the different response rates between countries. Especially in countries with low response rates, participating hospitals might comprise a selected group.

The present results are too preliminary to validate the proposed QI maturity scheme as an instrument. Our purpose was to classify hospitals in our sample for further analyses within the project according to their level of QI. Therefore we developed a scheme based on conceptual assumptions regarding QI in hospitals, and evaluated the sustainability of these assumptions statistically. Some of the results supported the concept of our maturity scheme, whereas others might be arguable from a psychometric point of view—for example, the high number of items, the very high Cronbach α for domains that were measured with large numbers of questionnaire items (eg, leadership), and the relatively low Cronbach α for accountability. However, the three independent validation analyses provide additional support for the validity of the classification scheme in our research sample.

It seems worthwhile to develop the current classification scheme further, into an instrument that can be used as a practical “quick scan” to assess the maturity of hospital QI systems. Once in place, it may help healthcare leaders at both the policy and the hospital level to identify areas on which to focus for further implementation of QI strategies. Bearing this in mind, further development of the maturity index classification into an instrument will require additional exploration and analyses of the MARQuIS study data to confirm our preliminary findings. Such analyses should include at least three actions. First, the maturity index should be simplified by deleting some items. Further statistical analysis would help to indicate which items discriminate least. Second, weighting of the domains requires further refinement. The domains that contribute to the overall maturity index should be identified, and their contributions should be translated into weightings that take into consideration issues such as the number of items to be included, among other aspects. Third, further reliability testing should be performed by applying the QI maturity index to other data sets.

## CONCLUSION

The proposed classification scheme, called here the maturity index, was useful in differentiating between hospitals in our research sample according to the maturity of their QI system, defined as the total of all QI activities. The validity of the results for our sample was supported by three different types of analysis. Further research is recommended to develop this scheme into an instrument that can be used as a practical “quick scan” to assess the maturity of hospital quality improvement systems.
